# Systemic Lupus Erythematosus Presenting With Severe Hypothyroidism and Extensive Pericardial Effusion in a Child

**DOI:** 10.7759/cureus.46172

**Published:** 2023-09-29

**Authors:** Rehab Aljasmi, Hadi Helali, Rasha Alloush, Boudour Khayer

**Affiliations:** 1 General Pediatrics, Al Jalila Children's Speciality Hospital, Dubai, ARE; 2 Pediatrics, Mohammed Bin Rashid University of Medicine and Health Sciences, Dubai, ARE; 3 Pediatric Emergency Medicine, Al Jalila Children's Speciality Hospital, Dubai, ARE

**Keywords:** autoimmune disease, periorbital edema, cardiac tamponade, pericardial effusion, hypothyroidism, systemic lupus erythematosus

## Abstract

Systemic lupus erythematosus (SLE) is a multisystem autoimmune disease, which may be associated with other autoimmune diseases, like autoimmune hypothyroidism. Both disorders can involve the cardiovascular system and cause pericardial effusion with cardiac tamponade. Herein, we describe a young eight-year-old female patient who initially presented with periorbital edema, cold intolerance, fatigue, and papular skin rash that was present on the face and the chest and was found to have significant pericardial effusion along with bilateral pleural effusion. Further laboratory investigation done in the hospital revealed severe hypothyroidism and positive SLE antibodies (antinuclear antibodies [ANA], antidouble strand DNA [anti-ds-DNA], and Sjögren’s syndrome antibodies A and B [SS-A and SS-B]). She was administered levothyroxine and pulse methylprednisolone, which significantly improved her condition. She was discharged on maintenance therapy with regular follow-ups with a multidisciplinary team.

## Introduction

Systemic lupus erythematosus (SLE) is a complex autoimmune condition characterized by multisystem involvement, which manifests as a wide range of clinical symptoms and signs as well as immunological abnormalities. It is more common in females with a female-to-male ratio of 8:1 in adults and 4:1 in pediatrics [1,2]. Although immunological, genetic, and environmental factors have been suggested as possible mechanisms, the exact etiology of SLE remains unknown [2]. In SLE, an initial aberrant immune response leads to the release of inflammatory cytokines and auto-generation of pathogenic autoantibodies. The inflammatory cytokines and autoantibodies cause end-organ inflammation and tissue damage [2]. Notably, SLE is a multisystem autoimmune disease that may affect various vital organ systems, including cardiovascular, renal, neurological (especially brain), mucocutaneous, musculoskeletal, and hematological systems [3].

It can also be associated with other organ-specific autoimmune diseases, such as autoimmune hypothyroidism [4]. Thyroid disease has been more frequently reported in patients with SLE than in the general population, particularly in cases with a higher incidence of antithyroid antibodies [4,5]. Hypothyroidism can present with a wide range of nonspecific symptoms such as fatigue, cold intolerance, weight gain, constipation, and, in severe cases, myxedema. The mechanism and manifestations of hypothyroidism on the cardiovascular system have been widely studied [6].

Additionally, pericardial diseases could result from hypothyroidism, including pericardial effusion, which has been reported on a large scale in many cases [7]. Conversely, SLE and pericardial effusion have been firmly linked [8]; however, links between the respective disease activities remain unknown [9]. So, both SLE and hypothyroidism can cause pericardial effusion independently, and both of them are associated with each other, which makes this case unique.

To the best of our knowledge, no case report in the current literature has demonstrated a simultaneous association of hypothyroidism and SLE to cardiac tamponade in the pediatric age group. In this report, we describe an eight-year-old patient who was diagnosed with significant pericardial effusion, severe hypothyroidism, and SLE at her first presentation.

## Case presentation

A previously healthy eight-year-old female patient presented to the emergency department (ED) with a three-month history of mild swelling around both eyes. Apart from mild breathing difficulty on exertion, she denied chest pain or palpitations. The patient reported weight gain of around 3 kg in the preceding month, constipation, and dryness of her skin. Furthermore, she experienced a generalized erythematous skin rash for a few weeks, especially in the chest, not sensitive to sunlight and not associated with fever. No family history of similar symptoms or autoimmune diseases was found. She was referred by her primary care doctor to be seen by the nephrologist in the hospital, who decided to refer her urgently to the ED after examining her.

Her clinical examinations in the ED revealed a body temperature of 36.5°C, heart rate of 82 beats per minute, respiratory rate of 20 breaths per minute, blood pressure of 119/92 mmHg, 100% oxygen saturation in room air, and capillary refill that was less than 2 seconds. Although she was well perfused, she was noted to have dry and cold skin with a mild erythematous macular rash in the trunk. Her eye movements were normal; however, mild edematous eyelids and mild periorbital edema were observed. She had no lower limb edema or thyroid enlargement on the neck exam. Her heart sounds were muffled without any additional sound (no murmurs and no clicks), and on lung auscultation, air entry was reduced bilaterally but was less on the right lung (especially lower lung field) in comparison to the left lung with no additional sounds (no wheezes or crepitation appreciated).

Investigations

**Figure 1:**
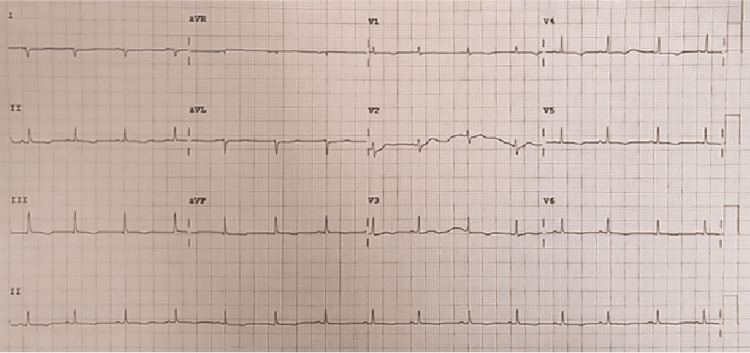
Electrocardiogram (ECG) showing diffuse low voltage in all leads

**Figure 2:**
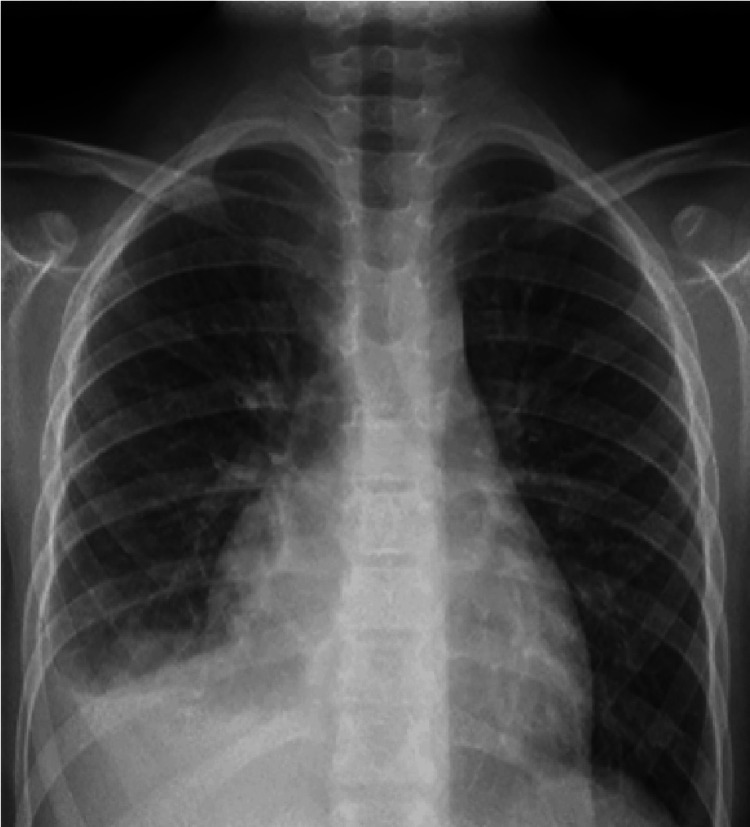
Chest radiograph showing mild cardiomegaly and right pleural effusion

**Figure 3:**
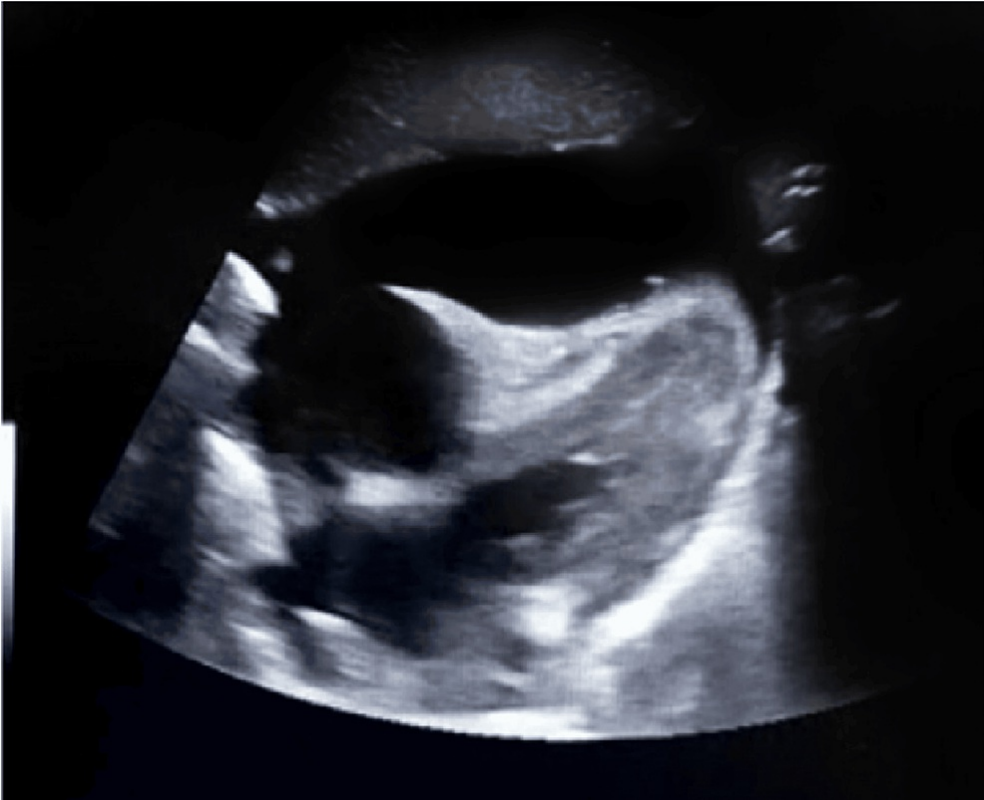
Point-of-care ultrasound: bedside echocardiography subcostal view with right ventricular collapse, which reflected significant pericardial effusion

Laboratory tests are documented in Tables 1, 2. The basic tests, including full blood counts (FBC), basic metabolic tests, and liver function tests (LFTs), were normal. The normal urine test, including urine dipstick and normal albumin level, excluded protein-losing pathology. An electrocardiogram (ECG) revealed generalized low voltage across all leads (Figure 1), suggesting pericardial effusion. A plain chest radiograph (Figure 2) disclosed mild right pleural effusion and cardiomegaly, which could be explained by massive pericardial effusion. This was confirmed using a bedside point-of-care echocardiogram in the ED (Figure 3).

**Table 1:** Initial laboratory investigations in emergency department CBC: Complete blood count; WBC: White blood cells; RBC: Red blood cells; HCT: Hematocrit; MCV: Mean corpuscular volume; MCH: Mean corpuscular hemoglobin; MCHC: Mean corpuscular hemoblogin concentration; RDW: Red cell distribution width; MPV: Mean platelet volume; ESR: Erythrocyte sedimentation rate.

Lab investigation	Results	Reference range
CBC		
WBC	8.53 x 10^3^/mcL	5-13 x 10^3^/mcL
RBC	8.53 x 10^6^/mcL	4-5.20 x 10^6^/mcL
Hgb	13.8 mg/dl	11.5-15.5 mg/dl
HCT	41.40%	35-45%
MCV	87.50 fL	77-95 fL
MCH	29.20 pg	25-33 pg
MCHC	33.30 mg/dl	31-73 mg/dl
RDW	17.40%	11.50-14.5%
Platelet	209 x 10^3^/mcL	170-450 x 10^3^/mcL
MPV	13.80 fL	8-9 fL
Neutrophil absolute	3.82 x 10^3^/mcL	2-8 x 10^3^/mcL
Lymph absolute	8.31 x 10^3^/mcL	1-5 x 10^3^/mcL
Monocyte absolute	1.44 x 10^3^/mcL	0.20-1 x 10^3^/mcL
Eos absolute	0.13 x 10^3^/mcL	0.10-1 x 10^3^/mcL
Basophil absolute	0.07 x 10^3^/mcL	0.02-0.1 x 10^3^/mcL
Basic metabolic		
Na	142 mmol/l	136-145 mmol/l
K	4.5 mmol/l	3.4-5.1 mmol/l
Cl	107 mmol/l	97-107 mmol/l
CO_2_	26 mmol/l	17-27 mmol/l
Creatinine	0.67 mg/dl	0.51-0.69 mg/dl
Urea	24 mg/dl	19.26-47.29 mg/dl
Inflammatory markers		
C-reactive protein	<1.0 mg/dl	0-5 mg/dl
ESR	7 mm/hr	0-10 mm/hr
Urine tests		
Urine creatinine	8.23 mg/dl	0-15 mg/dl

**Table 2:** Etiology-specific laboratory investigations in inpatient wards ALT: Alanine transaminase; AST: Aspartate aminotransferase; CGT: γ-glutamyltransferase; TSH: Thyroid-stimulating hormone; CK: Creatine kinase; LDH: Lactate dehydrogenase; ANA: Antinuclear antibodies; nRNP: Ribonucleoproteins; CENP B: Centromere protein B; PCNA: Proliferating cell nuclear antigen; AMA-M2: Antimitochondrial M2 antibody.

Lab investigation	Results	Reference range
Liver enzymes		
Protein	8.1 mg/dl	6.4-7.7 mg/dl
Albumin	5.1 g/dl	3.8-5.4 g/dl
Bilirubin total	0.41 mg/dl	0-1.20 mg/dl
Bilirubin direct	0.13 mg/dl	0-0.4 mg/dl
ALT	73 U/L	0-39 U/L
AST	71 U/L	0-51 U/L
Alkaline phosphate	88 IU/L	0-300 IU/L
GGT	26 U/L	0-31 U/L
Complement levels		
C3	40 mg/dl	82-172 mg/dl
C4	11.5 mg/dl	13-46 mg/dl
Thyroid function test		
TSH	199.9 mIU/ml	0.70-14.7 mIU/ml
T4	5.4 pmol/l	11.4-17.6 pmol/l
Cardiac profile		
CK	161 U/l	0-154 U/I
LDH	313 U/l	0-300 U/I
Troponin	<00.01 ng/ml	0 ng/ml
Antibodies profile		
ANA	Positive	Negative
nRNP/sm	Negative	Negative
SM	Negative	Negative
SS-A	Positive	Negative
SS-B	Positive	Negative
Ro-52	Negative	Negative
Scl-70	Negative	Negative
PM-Scl	Negative	Negative
Jo-1	Negative	Negative
CENP B	Negative	Negative
PCNA	Negative	Negative
Nucleosomes	Negative	Negative
Histones	Negative	Negative
AMA-M2	Negative	Negative
Anti-ds-DNA	Positive	Negative

Although inflammatory markers were normal, both compliments (C3 and C4) were found to be low (Table 2), whereas laboratory antinuclear antibodies (ANA) were positive. Furthermore, Sjögren’s syndrome (SS) antibodies both types A and B (SSA and SSB) and antidouble strand DNA (anti-ds-DNA) were positive. These laboratory tests and the clinical pictures of rash and serositis established the diagnosis of SLE.

Assessment of thyroid function tests showed extremely low levels of free thyroxine (5.4 pmol/L) and high levels of thyroid-stimulating hormone (199.4 mUI/L) (Table 2), which additionally diagnosed hypothyroidism.

**Figure 4:**
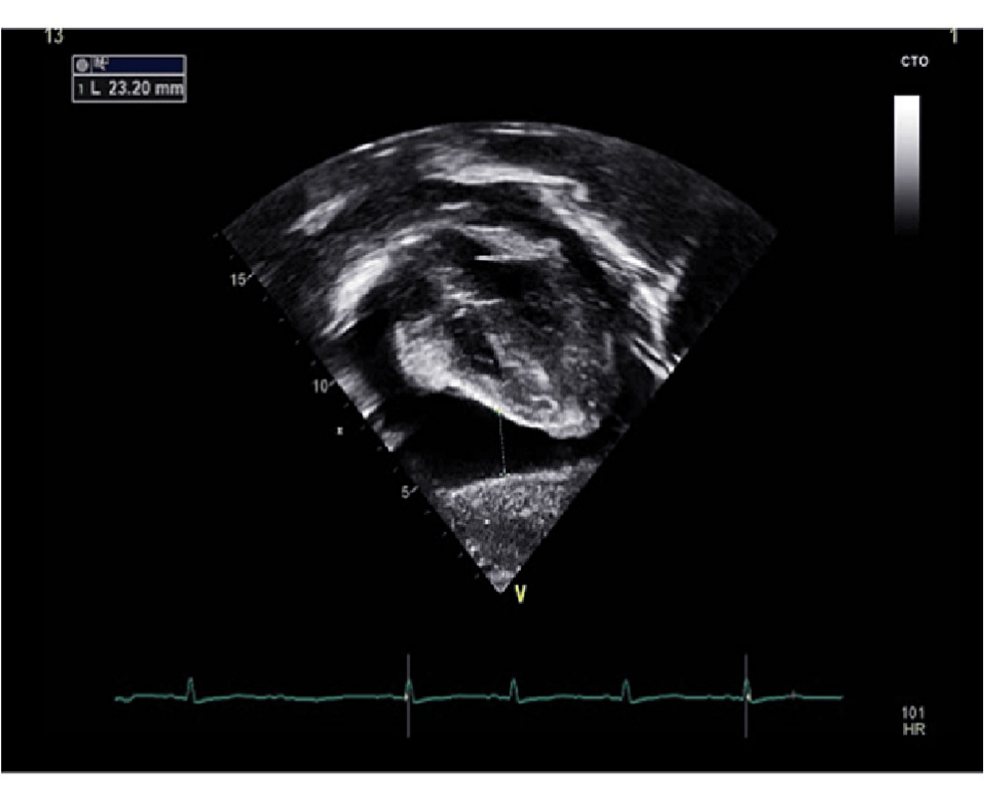
Echocardiogram demonstrating a large amount of pericardial effusion measures more than two centimeters

**Figure 5:**
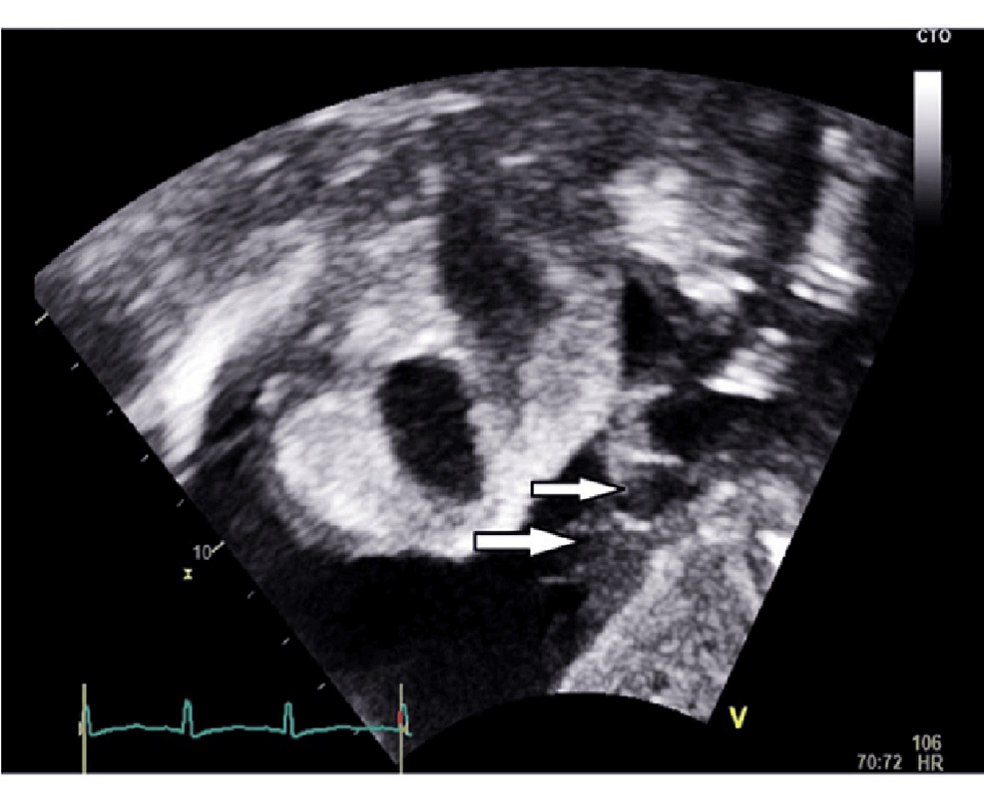
Echocardiogram showing the fibrin strands in the pericardial effusion (arrows) that represent the inflammatory process

An echocardiogram was performed by a pediatric cardiologist, which showed a large amount of pericardial effusion with a collapsing right atrium (Figure 4). Furthermore, the effusion contained some fibrin strands, although the pericardium was not thickened (Figure 5). Pericardiocentesis was performed under general anesthesia, and 300 mL of non-turbid, straw light yellow-colored fluid was drained. The pericardial fluid analysis showed 68 white blood cells (WBCs) in each milliliter (68 WBCs/ml) of 100% lymphocytes and did not grow any pathogen on gram staining and culture. *Mycobacterium tuberculosis* infection was excluded through negative results of acid-fast Bacilli staining of the pericardial fluid and fluid culture.

Treatment

The patient was managed by a multidisciplinary team including endocrinologists, rheumatologists, cardiologists, and general pediatricians. The patient was started on daily oral doses of 50 mcg of levothyroxine along with methylprednisolone 10 mg/kg/day for three days. An echocardiogram has been performed daily since her admission. On day 3, she developed pericardial fluid re-accumulation even after starting treatment with methylprednisolone and was drained for the second time, resulting in the removal of 150 mL of non-turbid, yellowish fluid with no blood. The pericardial tube drainage was removed on day 7 of admission. The initial methylprednisolone for three days was followed by daily oral administration of 1 mg/kg prednisolone and 6.5 mg/kg of hydroxychloroquine.

Outcome and follow-ups

The patient showed significant clinical improvement after starting the treatment, with marked improvement in energy since admission. Upon discharge, she resumed baseline activity, and the echocardiogram showed minimal pericardial effusion and normal cardiac function. The patient was discharged on oral prednisolone, hydroxychloroquine, and levothyroxine. After one month, she was observed in the rheumatology department and was asymptomatic on hydroxychloroquine and a weaning dose of prednisolone. Furthermore, she was observed in the endocrinology and cardiology departments after three months and showed well-controlled hypothyroidism and normal echocardiogram.

## Discussion

SLE affects people of all age groups; however, when it affects individuals < 18 years, it is identified as juvenile SLE (jSLE) or childhood-onset SLE (cSLE) [10].

Childhood-onset SLE (cSLE) differs from adult-onset SLE in several ways. First, the prevalence of SLE among children (1-6/100,000) is lower than that in adults (20-70/100,1000) [11]. Second, the female-to-male ratio in cSLE is 4-5:1, whereas it is 8-9:1 in SLE among adults [1]. Finally, although both are similar in pathogenesis, cSLE presents with a more severe course and diagnosis-challenging features [1]. As studies estimate, the period from presentation to diagnosis usually ranges from one month to 3.3 years because of the nonspecific and highly inconsistent initial symptomatic presentations in children with cSLE [12].

The cause of SLE is not yet completely understood; many factors might be involved such as genetic, hormonal, and environmental factors. Genetic factors include certain genetic abnormalities such as C1q, C2, and C4 deficiencies, and familial clustering suggests a non-Mendelian pattern of inheritance. Hormonal factors and the role of estrogen have been proposed based on the predominance of the disease in women of childbearing age. Environmental factors can include certain drugs, viruses, or ultraviolet light, which are linked to the activation of SLE [11].

The exact pathophysiology of SLE is yet to be defined. Certain environmental factors can induce physiological cell death, “apoptosis.” The suggested genetic factors may lead to failure in clearing the “apoptotic debris” in the bloodstream. These debris, which are mainly nuclear proteins, activate the immune system to generate autoantibodies targeting these nuclear proteins. Autoantibodies attack nuclear proteins in healthy tissues and form immune complexes (ICs). The deposition of ICs in the tissue results in tissue inflammation and the release of proinflammatory cytokines, mainly type I interferons (IFNα or INFβ). Type I interferons play a fundamental role in dendritic cell maturation and increase the inflammatory response [13].

Genetic factors play a significant role in determining the SLE age and phenotype, therefore determining cSLE. Defective genetic factors could affect any point of the autoimmunity process, including apoptosis dysregulation, immune cell activation, or interferon expression. Therefore, identifying these defects is crucial for the diagnosis and management planning of cSLE [14].

The general initial complaints in children diagnosed with cSLE are fever, weight loss, and malaise/fatigue, all of which are nonspecific and could be associated with multiple other diagnostic entities. However, due to the volatile nature of cSLE, not all children present with these symptoms altogether, and some of them (as in the case discussed above) may present with none of the above-mentioned symptoms or even with opposite ones [12].

The association between SLE and thyroid dysfunction has been established in the literature since 1961 [15]. In a recent review, Klionsky and Antonelli identified three risk factors for the development of hypothyroidism in patients with SLE: young age (<20 years), female sex, and severe SLE [16]. The etiology of these two diseases in concurrence is not well understood; however, two mechanisms were suggested: (i) immunological mechanism proposed by the high titer of antithyroglobulin in SLE patients and (ii) shared genetic structure for both diseases [16,17]. The association between SLE and hypothyroidism has been studied extensively in adults; however, few studies have addressed this among children [18,19].

Although pericardial effusion in small amounts is a recognized finding in pediatric patients with hypothyroidism, massive pericardial effusion is a rare complication [20]. However, the incidence of cardiac manifestations in patients with cSLE has not been well studied. The only large-group study (297 pediatric patients), performed by Chang et al. in the United States, showed that the incidence of cardiac manifestations in cSLE was higher and clinically worse than that in adult-onset SLE, with almost 19% presenting with cardiac disease almost a year before establishing SLE diagnosis. These conditions include myocarditis, pericarditis, and valvular involvement [21].

Multiple case reports have been published that resemble the case discussed above; however, they do not encompass its unique features and diagnostic challenges. Arabi et al. described two pediatric cases in their centers that presented with nonspecific symptoms and were later found to have cardiac tamponades. Further investigation revealed that they were also diagnosed with cSLE. Notably, these two cases were not associated with hypothyroidism [22].

Chaudhari et al. reported a case of an adult patient with SLE who developed symptoms suggestive of hypothyroidism and was later found to have massive pericardial effusion using echocardiography. However, this case was that of an adult patient who did not have concurrent SLE and hypothyroidism at the time of diagnosis with pericardial effusion, which makes the case discussed in this study unique in comparison [23]. A case similar to ours was published by Zhang et al. in an adult patient, whereas ours was a pediatric patient. This was further reflected in the fact that our patient did not complain of the same symptoms as theirs, but both were eventually diagnosed with SLE concurrent with hypothyroidism and pericardial effusion [4].

## Conclusions

The distinct features of childhood-onset SLE (cSLE) and its wide range of presentations could be challenging to diagnose, particularly if associated with other complications. Children presenting with hypothyroidism symptoms should be treated with intense care as they may have silent cardiac affection and other poly-autoimmunity-associated diseases, with cSLE being the most important. It is well known that severe and long-untreated hypothyroidism mimics rheumatologic diseases, especially JRA, Raynaud's phenomenon, and SLE. In these rheumatologic conditions, the patient must be evaluated for hypothyroidism.

Multidisciplinary teams are invaluable for managing similarly difficult cases. Children diagnosed with cSLE concurrent with hypothyroidism and cardiac affection need follow-up and management from pediatric rheumatology, endocrinology, and cardiology departments. Our case shows an example of successful integrated specialist teamwork that resulted in earlier diagnosis and appropriate management.
